# The Friend

**DOI:** 10.4269/ajtmh.20-1653

**Published:** 2021-08-02

**Authors:** Madhusudan Samprathi

**Affiliations:** Department of Pediatrics, All India Institute of Medical Sciences, Bibinagar, Hyderabad, Telangana, India

About 15 people were closeted in a small room, around a table. They sang a few hymns, their voices blending into a soft chorus. Then they opened their copies of a book, called “*Mythri*” (“friendship” in Sanskrit) and read short paragraphs one after another. The text was chosen from select verses of the Bible. It ended with a few questions for discussion on the relevance of those verses in daily life. By this time, a pastor had walked in to join the group at one end of the table to moderate the discussion.

The group was a mix of Christians, Hindus, and a Muslim. It was not a religious gathering or any place of worship. Yet, in few other places would God be remembered and his blessings invoked as much. For in the adjoining room, at least 10 children were fighting the toughest battles of their lives. For the distraught parents, life suddenly seemed to be an endless uncertain haze.

This unique session every Thursday morning is part of the weekly schedule at the pediatric intensive care unit (PICU) at the Christian Medical College in Vellore, India, one of the leading educational, healthcare, and research institutes in the country.

Like every Thursday morning, the PICU team, comprising of doctors, nurses, therapists, and ancillary staff sat together today for prayer and discussion, before the morning hand over. The pastor opened the session for discussion. The topic of the day was “work–life balance.” There was a minute of silence. One of the faculty set the ball rolling. How life had changed for her, from her days as a young trainee to a mature intensivist, with major milestones of becoming a wife and then a mother in the journey. How the stress of being an intensivist can take a toll at times, especially as an expectant mother, and later, with one’s own expectant children craving for their mother’s company. For me too, working in the ICU as a husband and father now, was dramatically more challenging than as a student a few years back.

The number and variety of roles played by each team member every single day can be mind boggling. I realized that my lady colleague in the ICU is also a mother who wakes up to her baby’s cry at dawn, a wife and homemaker, a daughter, a nurse taking care of a sick child of another mother, a trainee, and a researcher. All of these, every single day.

Though the social fabric and family dynamics are rapidly changing, Indian society has traditionally been considered largely patriarchal, with household chores being primarily the woman’s responsibility. I wondered how many men would work toward making a woman’s role easier. Not the role she has, as a wife, mother, or homemaker, but her role as a full-time professional. Not by limiting her opportunities at work, but by taking over some of her responsibilities at home.

The younger trainees slowly opened up. Like me, young and new parents had a lot to share, especially about the guilt of not being able to spend enough time with one’s family and children. I could see one trying to fight tears. Amid the hectic professional and academic life, one is still responsible for one’s own child whose childhood days will never return.

Senior members acknowledged that playing multiple demanding roles is an inevitable and continuing component of professional life, and empathy and understanding can smoothen the path. Some with work experience in western countries shared how having flexible work options and schedules can be a boon for new mothers.

It was heartening to see members senior to me realize the challenges and accept the limitations of the juniors, and humbling to see those much junior to me having more mature viewpoints.

After a few of these discussions, I was fascinated and started looking forward to Thursday mornings, to how verses from a religious text were recited to draw parallels to the current aspects of work and life. And how they could impact the daily life of the average person caring for critically ill children in the PICU.

Varied topics related to workplace (like achieving excellence, competition, hierarchy at work, gender discrimination, humiliation); patient care (like making mistakes, guilt, death, grief); and interpersonal relationships (like empathy, communication, professional jealousy) were discussed in an open, honest, lively, and interesting atmosphere. It was heartening to see thoughts, opinions, and emotions being put forth, cutting across designations and hierarchies, and more importantly, religions. How Christians could elaborate on the “Kingdom of God” and “grace,” in the same way a Hindu could speak on the “law of *Karma*.” In a way, we all gained from one another.

Intensive care is one of the most demanding specialities, a fact that we may not consciously and completely acknowledge while willingly opting for a lifetime in it. Being a physically exhausting, mentally tedious, emotionally draining, and intellectually challenging specialty, team work is paramount for achieving favorable patient outcomes. Intensive care unit teams are often heterogeneous with members from diverse backgrounds, and with varying capability, training, attitudes, strengths, and weaknesses. Work capacity, work output, critical thinking ability, enthusiasm, and energy, are all, like the patients’ physiology and disease, dynamic, and sometimes unpredictable. This makes the ICU working environment complex. Dealing with critically ill children and their families, declaring death, and withholding or withdrawing life support are events that can be extremely stressful at times. And given the number of patients and limited staff in developing countries, getting back to a normal frame of mind to care for the next sick child the next moment may not even seem possible or human.

Like the internal milieu of the patient, which we strive to understand and rectify day in and day out, the healer has an internal milieu too, which may not be in an ideal or optimal condition always, nor is possible to expect it to be. Personal and professional stresses, though two completely different spheres, ultimately act on one individual, and one mind. It may not be easy to recognize and reach out to team members on the verge of burnout, without falling prey to judgment, blame, or stigma. Understanding the temperament of the team members, the stresses that they face within and outside the unit and their reaction to these stresses, could uncover individual vulnerabilities. Recognizing these vulnerabilities could identify someone who needs help, and to offer help effectively in a nonjudgmental manner. Building and sustaining teams that effectively care for critically ill patients and empathetically deal with their families, cannot be done without caring for the carers and healing the healers.

Sessions like these can be valuable in understanding team members from a different and larger perspective, especially for the team leader. It may forge new relationships or open up new dimensions of familiar ones. Unraveling unique strengths may facilitate channelling them for better patient care. The beauty of team work is that individual flaws get nullified, and strengths multiply, so that patients get the best, even in the worst of circumstances, despite the complexity and uncertainty that pervades our profession, and especially, our specialty. All problems may not have solutions. But, acknowledging, accepting, and expressing a difficult emotion could itself be soothing and strengthening. For many of the trainees, being recognized and accepted as human, and fallible, with all one’s imperfections, but nevertheless, with the ability to do better, was in itself reassuring. Letting out can be the route to letting go.

Sometimes, these sessions took us back to where it all started, reminding us of the purpose with which we chose to be where we are. The purpose of all the research, academics, hectic schedules, and learning. Many things seemed to fall in place once the patient is the primary if not the sole focus of all that we do. Equally, if not more, important as enhancing knowledge and skills, is having a personality that can most meaningfully and effectively use them for what they are truly meant for. A challenging task, made relevant, simplified, and refreshing, by reposing every Thursday morning with a small book, which, like a true friend, helped us in letting out, and letting go.

